# Supplementary figures and images for: Growth inhibition associated with disruption of the actin cytoskeleton by Latrunculin A in rhabdomyosarcoma cells
Source: PLoS One. 2020 Sep 8;15(9):e0238572. doi: 10.1371/journal.pone.0238572 (PMC7478754; doi:10.1371/journal.pone.0238572)

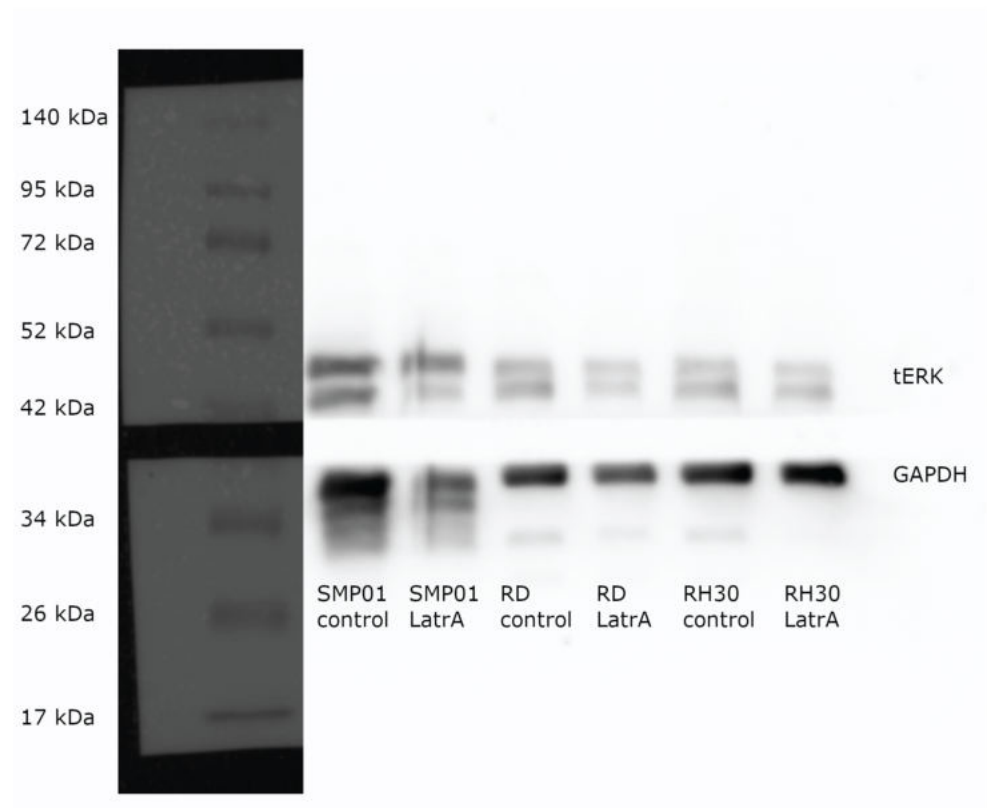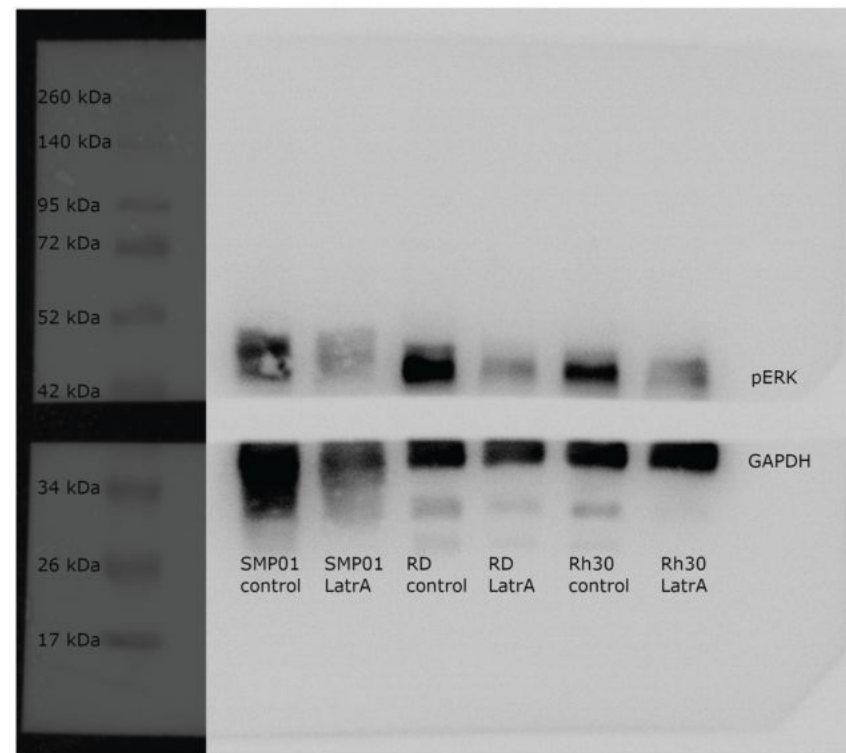

Supplement: S1 Raw images — Original uncropped and unadjusted images of the Western blots examining ERK1/2, p-ERK1/2 and GAPDH in RMS cells are provided. (PDF) [file pone.0238572.s001.pdf]

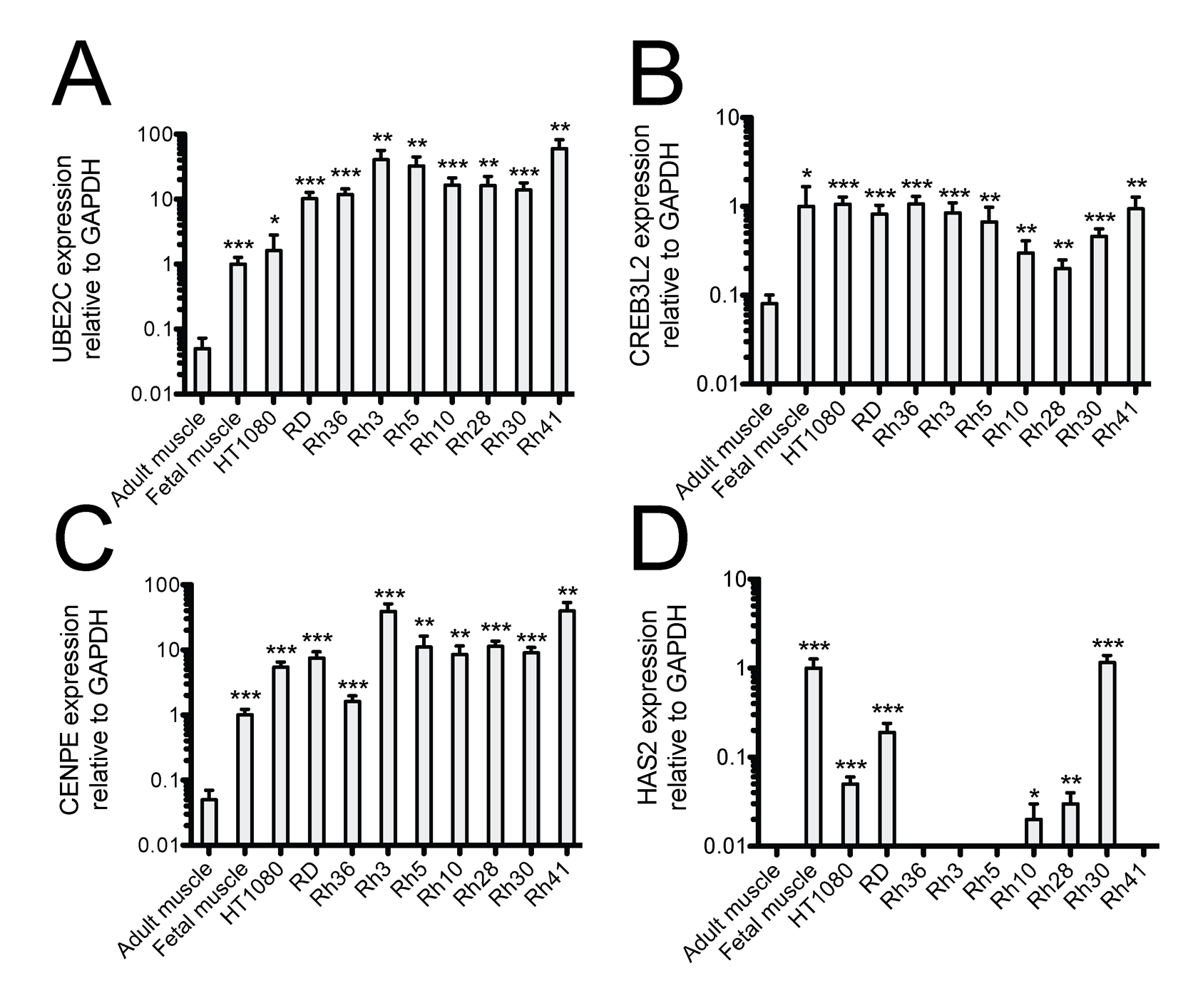

Supplement: S2 Fig — Expression of Cenpe, Has2, Creb3l2 and Ube2c in human sarcoma cell lines, as well as in human adult and human fetal muscle, was determined by qRT-PCR (Mean +/- SD of 4 technical replicates are presented; ns p≥0.05, * p<0.05, ** p<0.01, *** p <0.001, as determined by T-tests compared to adult muscle). (TIF) [file pone.0238572.s004.tif]
