# Supplementary material for: Growth inhibition associated with disruption of the actin cytoskeleton by Latrunculin A in rhabdomyosarcoma cells
Source: PLoS One. 2020 Sep 8;15(9):e0238572. doi: 10.1371/journal.pone.0238572 (PMC7478754; doi:10.1371/journal.pone.0238572)
Supplement: S1 Table — 141 sarcoma-relevant genes were identified by transcriptional profiling of KRAS-driven mouse sarcomas, and their contributions to sarcoma growth were probed by customized shRNA screening. Five candidate genes (marked in bold font) were found to be proliferation-relevant and immediately actionable [5, 6]. (DOCX) [file pone.0238572.s002.docx]

| **Gene symbol** | **Gene title** | **RefSeq** |
| --- | --- | --- |
| ADAM19 | ADAM metallopeptidase domain 19 (meltrin beta) | NM_009616 |
| ADAM8 | ADAM metallopeptidase domain 8 | NM_007403 |
| AGPAT4 | 1-acylglycerol-3-phosphate O-acyltransferase 4 (lysophosphatidic acid acyltransferase, delta) | NM_026644 |
| ALCAM | activated leukocyte cell adhesion molecule | NM_009655 |
| ARL4C | ADP-ribosylation factor-like 4C | NM_177305 |
| ARMCX2 | armadillo repeat containing, X-linked 2 | NM_026139 |
| **ASNS** | **asparagine synthetase** | **NM_012055** |
| BASP1 | brain abundant, membrane attached signal protein 1 | NM_027395 |
| BAZ1A | bromodomain adjacent to zinc finger domain, 1A | XM_885873 |
| BCAT1 | branched chain aminotransferase 1, cytosolic | NM_007532 |
| BTG1 | B-cell translocation gene 1, anti-proliferative | NM_007569 |
| CAND1 | cullin-associated and neddylation-dissociated 1 | XM_125901 |
| CAPG | capping protein (actin filament), gelsolin-like | NM_007599 |
| CCDC109B | coiled-coil domain containing 1 | NM_025779 |
| CCNA2 | cyclin A2 | NM_009828 |
| CCND1 | cyclin D1 | NM_007631 |
| CDC20 | cell division cycle 20 homolog (S. cerevisiae) | NM_023223 |
| CDCA4 | cell division cycle associated 4 | NM_028023 |
| CDH11 | cadherin 11, type 2, OB-cadherin (osteoblast) | NM_009866 |
| CDH2 | cadherin 2, type 1, N-cadherin (neuronal) | NM_007664 |
| CDK5R1 | cyclin-dependent kinase 5, regulatory subunit 1 (p35) | NM_009871 |
| CDK6 | cyclin-dependent kinase 6 | NM_009873 |
| CDT1 | chromatin licensing and DNA replication factor 1 | NM_026014 |
| CENPA | centromere protein A | NM_007681 |
| **CENPE** | **centromere protein E, 312kDa** | **NM_173762** |
| CEP55 | centromere protein E, 312kDa | NM_028760 |
| CHSY1 | chondroitin sulfate synthase 1 | NM_001081163 |
| CKLF | chemokine-like factor | NM_029295 |
| CKS1B | CDC28 protein kinase regulatory subunit 1B | NM_016904 |
| CKS2 | CDC28 protein kinase regulatory subunit 2 | NM_001827 |
| CLCN5 | chloride channel 5 | NM_016691 |
| COL18A1 | collagen, type XVIII, alpha 1 | NM_009929 |
| COTL1 | coactosin-like 1 (Dictyostelium) | NM_028071 |
| **CREB3L2** | **AMP responsive element binding protein 3-like 2** | **NM_178661** |
| CSRP2 | cysteine and glycine-rich protein 2 | NM_007792 |
| CTTN | cortactin | NM_007803 |
| CYBA | cytochrome b-245, alpha polypeptide | NM_007806 |
| DAP | death-associated protein | NM_146057 |
| DBF4 | DBF4 homolog (S. cerevisiae) | NM_013726 |
| DBN1 | drebrin 1 | NM_019813 |
| DOK1 | docking protein 1, 62kDa (downstream of tyrosine kinase 1) | NM_010070 |
| EDEM1 | ER degradation enhancer, mannosidase alpha-like 1 | NM_138677 |
| EDNRA | endothelin receptor type A | NM_010332 |
| EFHD2 | EF-hand domain family, member D2 | NM_025994 |
| EGR2 | early growth response 2 | NM_010118 |
| EGR3 | early growth response 3 | NM_018781 |
| ETV1 | ets variant 1 | NM_007960 |
| EZR | ezrin | NM_009510 |
| F2RL1 | coagulation factor II (thrombin) receptor-like 1 | NM_007974 |
| FKBP11 | FK506 binding protein 11, 19 kDa | NM_024169 |
| FN1 | Fibronectin | NM_010233 |
| FRK | fyn-related kinase | NM_010237 |
| FSCN1 | fascin homolog 1, actin-bundling protein (Strongylocentrotus purpuratus) | NM_007984 |
| GAL | galanin prepropeptide | NM_010253 |
| GALNS | galactosamine (N-acetyl)-6-sulfate sulfatase | NM_016722 |
| GAS2L1 | growth arrest-specific 2 like 1 | NM_144560 |
| GEM | GTP binding protein overexpressed in skeletal muscle | NM_010276 |
| GJC1 | gap junction protein, gamma 1, 45kDa | NM_008122 |
| GLIPR1 | GLI pathogenesis-related 1 | NM_028608 |
| GNB1 | guanine nucleotide binding protein (G protein), beta polypeptide 1 | NM_008142 |
| GPR137B | G protein-coupled receptor 137B | NM_031999 |
| GREM1 | gremlin 1, cysteine knot superfamily, homolog (Xenopus laevis) | NM_011824 |
| GSTCD | glutathione S-transferase, C-terminal domain containing | NM_026231 |
| **HAS2** | **hyaluronan synthase 2** | **NM_008216** |
| HELLS | helicase, lymphoid-specific | NM_008234 |
| HMGB2 | high-mobility group box 2 | NM_008252 |
| HMGCR | 3-hydroxy-3-methylglutaryl-Coenzyme A reductase | NM_008255 |
| IER3 | immediate early response 3 | NM_133662 |
| KDELR2 | KDEL (Lys-Asp-Glu-Leu) endoplasmic reticulum protein retention receptor 2 | NM_025841 |
| LASP1 | LIM and SH3 protein 1 | NM_010688 |
| LBH | mb bud and heart development homolog (mouse) | NM_029999 |
| LRRFIP1 | leucine rich repeat (in FLII) interacting protein 1 | NM_008515 |
| MARCKSL1 | MARCKS-like 1 | NM_010807 |
| MCM6 | minichromosome maintenance complex component 6 | NM_008567 |
| MPZL1 | myelin protein zero-like 1 | NM_001001880 |
| MYO1B | myosin IB | NM_010863 |
| MYO9B | myosin IXB | NM_015742 |
| NCAM1 | neural cell adhesion molecule 1 | NM_010875 |
| NGFRAP1 | nerve growth factor receptor (TNFRSF16) associated protein 1 | NM_009750 |
| NMT2 | N-myristoyltransferase 2 | NM_008708 |
| NR2F1 | Nuclear receptor subfamily 2, group F, member 1 | NM_010151 |
| PAFAH1B3 | platelet-activating factor acetylhydrolase, isoform Ib, subunit 3 (29kDa) | NM_008776 |
| PBK | PDZ binding kinase | NM_023209 |
| PDLIM4 | PDZ and LIM domain 4 | NM_019417 |
| PDLIM5 | PDZ and LIM domain 5 | NM_019808 |
| PHF16 | PHD finger protein 16 | NM_199317 |
| PHGDH | phosphoglycerate dehydrogenase | NM_016966 |
| PHLDA1 | pleckstrin homology-like domain, family A, member 1 | NM_009344 |
| PLK4 | polo-like kinase 4 (Drosophila) | NM_011495 |
| POLD3 | polymerase (DNA-directed), delta 3, accessory subunit | NM_133692 |
| PRC1 | protein regulator of cytokinesis 1 | NM_145150 |
| PRIM2 | primase, DNA, polypeptide 2 (58kDa) | NM_008922 |
| PRKG2 | protein kinase, cGMP-dependent, type II | NM_008926 |
| PSAT1 | phosphoserine aminotransferase 1 | NM_177420 |
| PTGES | prostaglandin E synthase | NM_022415 |
| PTGS1 | prostaglandin-endoperoxide synthase 1 (prostaglandin G/H synthase and cyclooxygenase) | NM_008969 |
| PTK2B | PTK2B protein tyrosine kinase 2 beta | NM_172498 |
| PTPRG | protein tyrosine phosphatase, receptor type, G | NM_008981 |
| RAD54L | RAD54-like (S. cerevisiae) | NM_009015 |
| RAI14 | retinoic acid induced 14 | NM_030690 |
| RASA2 | RAS p21 protein activator 2 | NM_053268 |
| RBBP8 | retinoblastoma binding protein 8 | NM_001081223 |
| RBP1 | retinol binding protein 1, cellular | NM_011254 |
| RDH11 | retinol dehydrogenase 11 (all-trans/ 9-cis/ 11-cis) | NM_021557 |
| RGS19 | regulator of G-protein signaling 19 | NM_026446 |
| RUNX1 | runt-related transcription factor 1 | NM_009822 |
| SC4MOL | sterol-C4-methyl oxidase-like | NM_025436 |
| SDC1 | syndecan 1 | NM_011519 |
| SDC2 | syndecan 2 | NM_008304 |
| SH3BGRL3 | SH3 domain binding glutamic acid-rich protein like 3 | NM_080559 |
| SHCBP1 | SHC SH2-domain binding protein 1 | NM_011369 |
| SIPA1L3 | signal-induced proliferation-associated 1 like 3 | NM_001081028 |
| SKP2 | S-phase kinase-associated protein 2 (p45) | NM_013787 |
| SLC20A1 | solute carrier family 20 (phosphate transporter), member 1 | NM_015747 |
| SLC25A24 | solute carrier family 25 (mitochondrial carrier; phosphate carrier), member 24 | NM_172685 |
| SLC2A1 | solute carrier family 2 (facilitated glucose transporter), member 1 | NM_011400 |
| SLC39A6 | solute carrier family 39 (zinc transporter), member 6 | NM_139143 |
| SLC5A3 | solute carrier family 5 (sodium/myo-inositol cotransporter), member 3 | NM_017391 |
| SMC2 | structural maintenance of chromosomes 2 | NM_008017 |
| SNAI2 | snail homolog 2 (Drosophila) | NM_011415 |
| SOCS5 | suppressor of cytokine signaling 5 | NM_019654 |
| SOX11 | SRY (sex determining region Y)-box 11 | NM_009234 |
| SOX9 | SRY (sex determining region Y)-box 9 | NM_011448 |
| SPC25 | SPC25, NDC80 kinetochore complex component, homolog (S. cerevisiae) | NM_025565 |
| SQLE | squalene epoxidase | NM_009270 |
| TES | testis derived transcript (3 LIM domains) | NM_011570 |
| TGFB1I1 | transforming growth factor beta 1 induced transcript 1 | NM_009365 |
| TGIF1 | TGFB-induced factor homeobox 1 | NM_009372 |
| TIMP1 | TIMP metallopeptidase inhibitor 1 | NM_011593 |
| TMEFF1 | transmembrane protein with EGF-like and two follistatin-like domains 1 | NM_021436 |
| TNC | tenascin C | NM_011607 |
| TOP2A | topoisomerase (DNA) II alpha 170kDa | NM_011623 |
| TPBG | trophoblast glycoprotein | NM_011627 |
| TRIP13 | thyroid hormone receptor interactor 13 | NM_027182 |
| TUBA1A | tubulin, alpha 1a | NM_011653 |
| TUBB2A | tubulin, beta 2A | NM_009450 |
| TUBB6 | tubulin, beta 6 | NM_026473 |
| **UBE2C** | **ubiquitin-conjugating enzyme E2C** | **NM_026785** |
| UNC5B | Unc-5 homolog B (C. elegans) | NM_029770 |
| VCAN | versican | NM_001081249 |
| ZWINT | ZW10 interactor | NM_025635 |
