# Supplementary material for: Growth inhibition associated with disruption of the actin cytoskeleton by Latrunculin A in rhabdomyosarcoma cells
Source: PLoS One. 2020 Sep 8;15(9):e0238572. doi: 10.1371/journal.pone.0238572 (PMC7478754; doi:10.1371/journal.pone.0238572)
Supplement: Attachment — Submitted filename: Response to reviewers_2020 07 02.docx [file pone.0238572.s007.docx]

Detailed point-by-point response:

Responses are marked in blue. Changes were marked using the track changes function in the revised manuscript.

Reviewer #1: All the comments have been addressed by the authors. The paper is now worth of publication without further modifications.

We thank the reviewer for the positive feedback.

Reviewer #2: There are few typos, for example in the methods section, paragraph "Customized shRNA proliferation screen": " T-tests and the algorithm published by the algorithm published by J. W.McNicol and G. Hogan (11)."

We apologize for the typos. They were corrected.

Moreover I cannot find the " Supplemental Experimental Procedures ", please contact the editor.

To provide readers with easier access to the technical details, all experimental procedures were included within the main manuscript. The reference to Supplemental Experimental Procedures was removed. We apologize for placing this misleading sentence in the previous versions of the manuscript.
